# A Novel Single-Axis MEMS Tilt Sensor with a High Sensitivity in the Measurement Range from 0^∘^ to 360^∘^ †

**DOI:** 10.3390/s18020346

**Published:** 2018-01-25

**Authors:** Shudong Wang, Xueyong Wei, Yinsheng Weng, Yulong Zhao, Zhuangde Jiang

**Affiliations:** State Key Laboratory for Manufacturing Systems Engineering, Xi’an Jiaotong University, Xi’an 710054, China; shudong_wang@outlook.com (S.W.); wengyinsheng@stu.xjtu.edu.cn (Y.W.); zhaoyulong@mail.xjtu.edu.cn (Y.Z.); zdjiang@mail.xjtu.edu.cn (Z.J.)

**Keywords:** double-ended tuning fork, MEMS, oscillator, resonator, tilt sensor

## Abstract

In this paper, a novel single-axis MEMS tilt sensor is presented. It contains a hexagonal proof mass, six micro-lever force amplifiers and three double-ended-tuning fork (DETF) resonant strain gauges. The proof mass is placed in the center with the micro-levers and the DETFs radially arrayed around. The variation of gravity acceleration applied on the proof mass will result in frequency shifts of the DETFs. Angular tilt can be got by analyzing the frequency outputs. The structural design of the tilt sensor is optimized by finite element simulation and the device is microfabricated using a silicon-on-insulator process, followed by open-loop and closed-loop characterizations. Results show that the scale factor of such sensor is at least 11.53 Hz/degree. Minimum Allan deviation of the DETF oscillator is 220 ppb (parts per billion) of the resonant frequency for an 5 s integration time. Resolution of the tilt sensor is $0.002{}^{\circ }$ in the whole measurement range from $0{}^{\circ }$ to $360{}^{\circ }$.

## 1. Introduction

Accurate tilt measurement is of great significance in many areas of civil, industrial and military uses. It has been utilized in somatic games, remote health monitoring for large structures, inertial navigations and etc. Therefore, many research groups are devoted to improve the performance of tilt sensors and related measuring systems [1,2,3]. Particularly, since the rapid development of MEMS technology, many kinds of sensors have been revolutionized and enabled a smaller dimension, a better performance and a lower price, leading to a rapid increase in the market share of sensors based on MEMS technology.

The main concept behind these MEMS tilt sensors is almost the same. First, there is a movable proof mass to sense the inertial force caused by gravity. This mass could be solid [4,5], liquid [6] or gas. Then there must be a physical response to the moving of the mass, which could be the change of capacitance [7], resistance [8] or resonance frequency [9,10,11,12]. An external circuit is needed to convert these responses to electrical signal, and angular tilt can be got through processing the electrical signal. Among those above, resonant MEMS tilt sensors are more attractive due to their quasi-digital output signal and high accuracy. Plenty of work have been devoted to improve the reliability and sensitivity of the resonant MEMS tilt sensors. Using differential structure is a common method to reduce the interference of temperature and residual stress [13]. One-stage or multi-stage micro-levers were employed and proved to be effective ways to amplify the inertial force as well as the sensitivity of tilt sensors [14,15]. In addition, mode localization effect in coupled micromechanical resonators was studied in MEMS and became a new sensing mechanism for tilt sensors [16]. Recently, a tilt sensor with a scale factor of 15.5 Hz/degree maintained in $\pm 20{}^{\circ }$ was reported, which is the highest among all [9]. Nevertheless, it is still hard to achieve a high sensitivity in a larger measurement range because of the sensor design and the related sensing mechanism. In this work, a novel MEMS tilt sensor is proposed to overcome the limited measurement range of high sensitivity. The sensing mechanism and structural design are first introduced, followed by open-loop and closed-loop characterizations. The experimental results indicate that the tilt sensor can maintain a high sensitivity in the whole measurement range from $0{}^{\circ }$ to $360{}^{\circ }$.

## 2. Sensing Principle and Structure Design

### 2.1. Sensing Principle

When a tilt angle is applied, sensing element of the tilt sensor will be affected by the gravity component, leading to a physical response, from which the tilt can be derived. In the case of MEMS resonance sensor, this physical response is frequency shift. Due to the direction of the gravity is always vertical to the ground, the physical response versus angle presents a sine function, causing a different sensitivity in different position.

**Figure 1:**
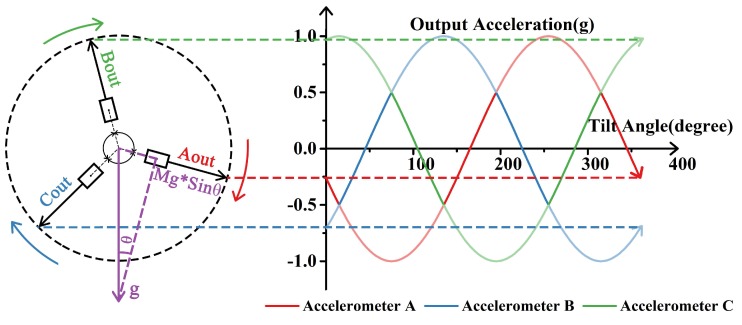
Schematic drawing of tilt measurement based on three accelerometers and simulated output of each accelerometer as rotating.

Thus, tilt sensor made by a single element will have a limited measurement range of high sensitivity, generally $\pm 30{}^{\circ }$, in which the relation between tilt angle and output acceleration is approximately linear [17]. However, the range will be extended using a combination of three sensing elements, as shown in Figure 1. When three sensing elements are radially arranged with $120{}^{\circ }$ apart from each other, there is always one sensing element more sensitive than the other two. Using data of the most sensitive element at any position, an accurate measurement of tilt in the whole range will be achieved accordingly. Łuczak gave the concept of tilt measurement systems utilizing multi-axial accelerometers [18], and disscussed application of arc tangent function, which can also ensure a constant sensitivity of tilt measurement [19]. Enfu Li et al. achieved a tilt sensor with a high resolution maintained in the full measurement range based on one dual-axis micro-accelerometer [20]. In our previous work, we built a tilt measurement system based on three accelerometers, whose resolution is $0.02{}^{\circ }$ [3]. In this work, we will integrate three sensing elements in MEMS process, and propose a MEMS tilt sensor with a smaller size, and a better performance.

### 2.2. Sensor Design

**Figure 2:**
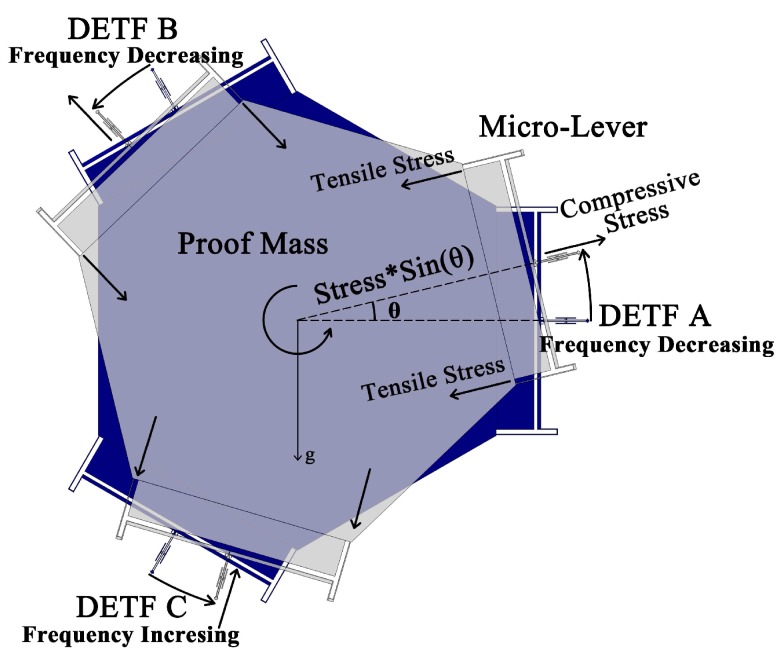
Structure of the tilt sensor.

Based on aforementioned sensing principle, a novel single-axis MEMS tilt sensor is thus proposed as shown in Figure 2. It contains a hexagon proof mass, six micro-lever force amplifiers and three DETFs. The proof mass is placed in the middle with the micro-levers and the DETFs radially arrayed around, with a $120{}^{\circ }$ apart from each other, as discussed above. All of the DETFs are connected to the proof mass through the micro-levers. Under working conditions, the tilt sensor is placed in the vertical plane. When the proof mass displaces due to the gravity, different components of gravity will apply on each micro-lever, which amplifies the force exerted on the DETFs. As a result, the resonant frequencies of DETF resonators will be tuned [12] and can be expressed as follow:

(1) $$\begin{array}{c} f=f_{n}\sqrt{1+\frac{0.293Pl^{2}}{Etw^{3}}} \end{array}$$

where $f_{n}$ is the characteristic frequency of the DETF, *l*, *w* and *t* indicate the length, width and thickness of each resonant beam respectively. *P* is the axial force applied on the resonant beam, which is a negative number when compressive stresses applied and it is a positive number when tensile stresses applied, *E* represents the Young’s modulus of silicon.

Method of finite element analysis was used to aid the design of the structure and find the optimum dimensions. Relationship between frequency shift and tilt angle can be easily botained as well in the simulation. Accordingly, the scale factor of tilt sensor is calculated by:

(2) $$\begin{array}{c} S=\frac{\Delta f}{\Delta \theta } \end{array}$$

where Δf is the variation of DETF’s resonant frequency and Δθ is the inclination of the whole device. Note that scale factor is a variate according to the angular position, and it remains high-level in ±30°, which is also called “sensitive region”.

**Figure 3:**
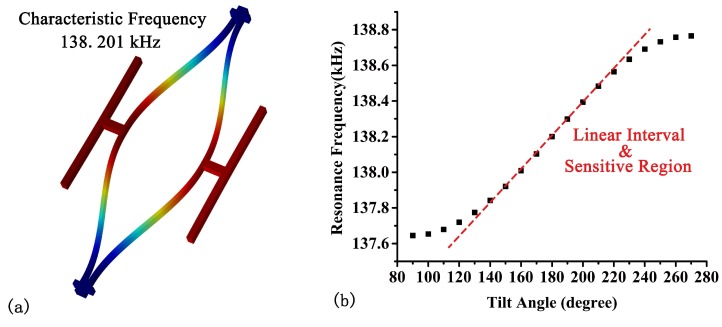
Vibration mode of DETF B (**a**), and its resonant frequency versus inclination (**b**).

In order to maximize Δf, six micro-lever force amplifiers were used to magnify the force applied on each DETF. The micro-lever conprises an input beam, a lever beam, a pivot beam and an output beam [21]. The input beam was designed to be long and in folded form, thus minimize the effect of the component gravitational acceleration perpendicular to the axis of the DETFs, also known as “crosstalk”. The optimized geometry dimensions of the tilt sensor are given in Table 1. Figure 3 shows the vibration mode of DETF B (a), and the simulation results of its resonant frequency versus inclination (b). The approximate linear interval and sensitive region is marked in the figure.

Due to the anisotropy properties of single-crystal-silicon being used, two of the DETFs are of the same resonant frequency due to their similar crystal orientation on the silicon wafer, while the other one is far different. Thus a normalization of their frequencies is required to identify the DETF that is in the most sensitive region:

(3) $$\begin{array}{c} f_{norm}=\frac{f_{M}-f_{avg}}{\frac{1}{2}f_{pp}} \end{array}$$

where, $f_{M}$ is the measured frequency of each DETF, $f_{avg}$ is the average of the frequencies in the whole measurement range, $f_{pp}$ is the peak-to-peak value of frequencies.

After normalization, frequencies of the three DETFs will transform into three standard sine waves, as shown in Figure 1, and the most sensitive DETF at each given position can be clearly found out. Thus, the angle θ can be got and the formula is:

(4) $$\begin{array}{c} \theta =sin^{-1}\left(f_{norm}\right) \end{array}$$

where $f_{norm}$ is the normalized output frequency of the most sensitive one of the three DETFs at the given position.

Detailed information was given in our previous work [21]. As a result, the simulated sensitivities of DETF A, B and C are 10.38 Hz/degree, 9.34 Hz/degree and 9.33 Hz/degree respectively. Therefore, the scale factor of the sensor in the measurement range from $0{}^{\circ }$ to $360{}^{\circ }$ is theoretically no less than 9.33 Hz/degree.

### 2.3. Temperature Drift

**Figure 4:**
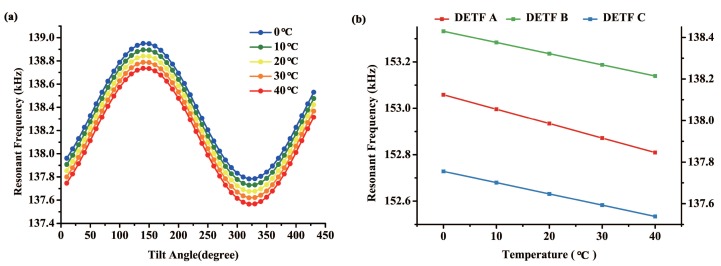
Resonant frequency of DETF B in different temperature environments vs. tilt angle (**a**) and resonant frequency of the three DETFs at a given position vs. temperature (**b**).

Single-crystal silicon is an anisotropic material whose elasticity matrix and Young’s modulus change as temperature varies. In this way, effect of external temperature should be considered and compensated. In order to quantify this effect, finite element analysis method was used and the results are shown in Figure 4. Thermal expansion was assumed to be negligible in this simulation model.

Figure 4a demonstrates resonant frequency of DETF B in different temperature environments versus tilt angle. As the temperature rises, characteristic frequency of the DETF reduces, while the scale factor remains the same. Figure 4b shows the linear relation between resonant frequency and ambient temperature. The fitted slopes of the three lines are 6.21 Hz/${}^{\circ }$C, 5.4 Hz/${}^{\circ }$C, and 5.4 Hz/${}^{\circ }$C respectively. The differences among them may caused by different crystal orientations.

Temperature drift will not affect scale factor or resolution of the tilt sensor, however, it will reduce stability of the frequency data and effect the result of angle calculation. Therefore, in practical use, influence of external temperature should be compensated [9,22].

## 3. Experimental Results of Open-Loop Test

**Figure 5:**
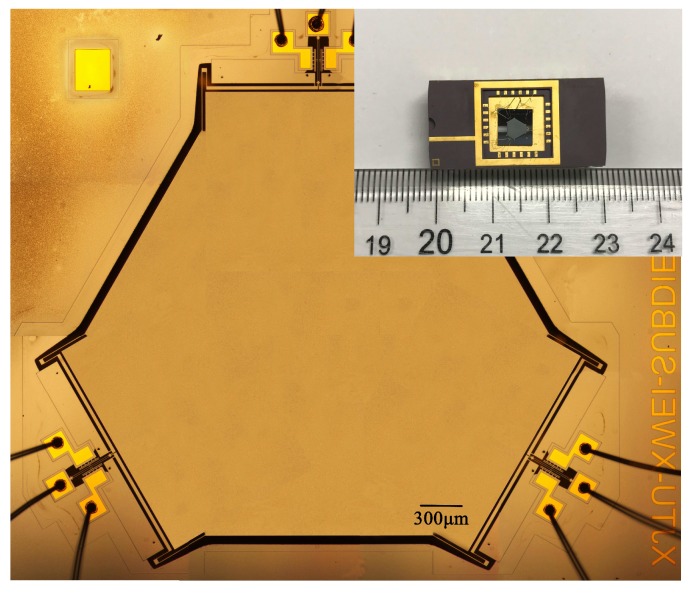
Micrograph of the tilt sensor and package of the device.

The described tilt sensor was microfabricated using a standard SOI-MEMS technology and the mechanical layer thickness of the SOI is 10 μm. A micrograph of the tilt sensor and its package is shown in Figure 5. Three DETF resonators are separated $120{}^{\circ }$ apart and connect to a common proof mass in the center through three pairs of lever amplifiers.

**Figure 6:**
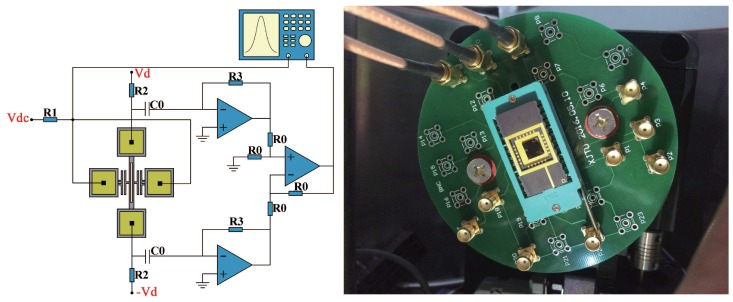
Schematic diagram of the open-loop test circuit (**a**) and installation (**b**).

Figure 6 shows the schematic diagram of the open-loop test circuit (a) and the testing setup (b). The tilt sensor was mounted in the vertical plane on a rotary table, which is placed in a vacuum chamber. The rotary table was operated by an external controller, and its positionaI accuracy was 0.01°. The vacuum level was settled to 10 Pa thus the effect of air damping was reduced and a high Q-factor of the DETF was achieved. When the rotary table rotates, the three DETFs are subject to different component gravitational acceleration as illustrated in Figure 2. 

**Figure 7:**
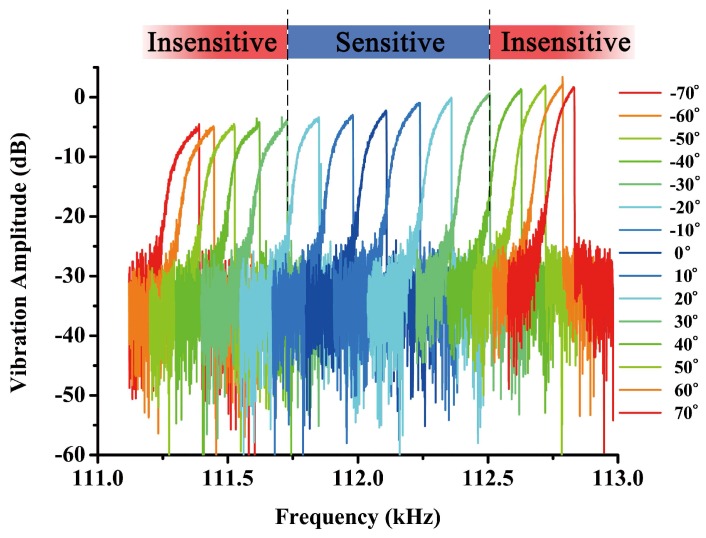
Open-loop test result of DETF B from $-50{}^{\circ }$ to $50{}^{\circ }$.

In this experiment, the DETF resonator is electrically driven and sensed through parallel electrodes. A DC source (KEYSIGHT E3649A) was used to provide DC-bias on the electrodes. A network analyzer (KEYSIGHT E5061B) was also applied to characterize the frequency responses of the three DETFs. A differential piezoresistive sensing scheme was used to achieve a better feedthrough cancellation and a higher signal-noise ratio [23]. Figure 7 shows the vibration amplitude of DETF B obtained from the network analyzer when the device is tilted from $-70{}^{\circ }$ to $70{}^{\circ }$ at a step of $10{}^{\circ }$. The resonant frequency of DETF B is tuned as the rotary table rotates, and the frequency shift is larger within the range from $-30{}^{\circ }$ to $30{}^{\circ }$, which is the sensitive region of DETF B as indicated by Figure 1.

The frequency response curves in Figure 7 indicate that the DETF is oscillating in its nonlinear vibration state. As the driving frequency sweeps upward and downward, a hysteresis region will be observed with two jumping points in the frequency response curves [24]. Both frequencies at the jumping points are subject to axial stress tuning of DETF. In the experiment, we found that the frequency at the lower jumping point is more stable than the peak frequency at the upper jumping point as the sensor tilts. Therefore, the frequency data was recorded while the rotary table rotates from $0{}^{\circ }$ to $360{}^{\circ }$ with a step of $10{}^{\circ }$ and the experiment was done in a rather stable vacuum pressure and ambient temperature.

**Figure 8:**
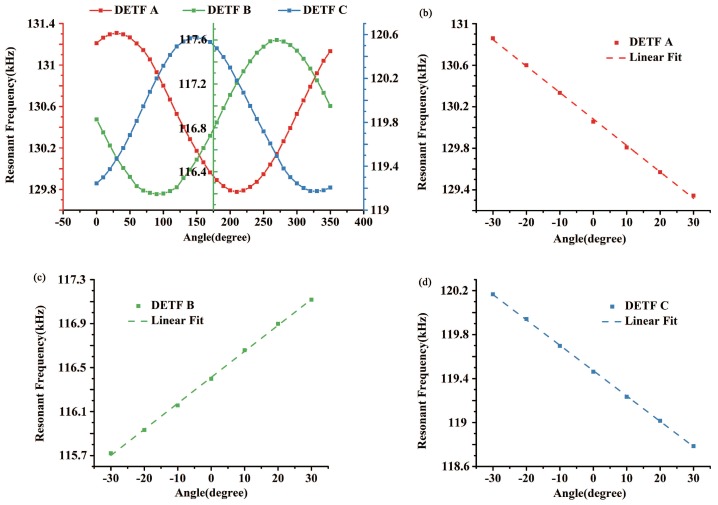
(**a**) Resonant frequency of the three DETFs got by downward sweeps; (**b**) Frequency of DETF A in sensitive region; (**c**) Frequency of DETF B in sensitive region; (**d**) Frequency of DETF C in sensitive region.

Figure 8a shows the resonant frequencies of three DETFs versus tilt angle and accordingly there are three sine curves of frequencies with phase differences of $120{}^{\circ }$ as predicted in Figure 1. Due to the anisotropic properties of single crystal silicon and the unavoidable processing error, the characteristic frequencies of the micro-fabricated DETFs slightly differ from the FEA simulation results and vary from each other. The frequency response of each DETF in the sensitive region is extracted and shown in Figure 8b, c and d respectively. After a linear fitting of the data, the scale factors of three DETFs are 12.73 Hz/degree, 11.84 Hz/degree and 11.53 Hz/degree respectively. Therefore, the scale factor of the tilt sensor is no less than 11.53 Hz/degree.

**Figure 9:**
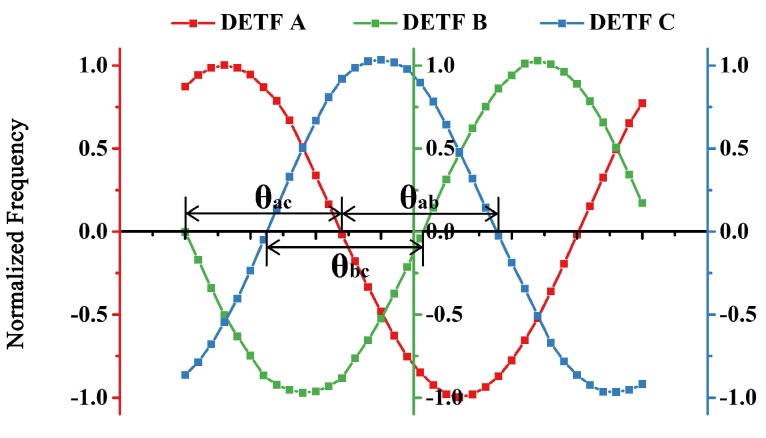
Normalized Frequency of the three DETFs.

Three DETFs were designed to be $120{}^{\circ }$ apart from each other, however, the angles between each DETF are slightly different from $120{}^{\circ }$ due to manufacturing process error. These angular misalignments can be obtained by analyzing the normalized frequency of the three DETFs derived from (5). As Figure 9 shows, after measuring and fitting the data, it was found that θab is $119.8{}^{\circ }$, θbc is $119.54{}^{\circ }$, and θac is $120.66{}^{\circ }$. These errors will be considerded in the future analysis.

## 4. Experimental Results of Closed-Loop Test

**Figure 10:**
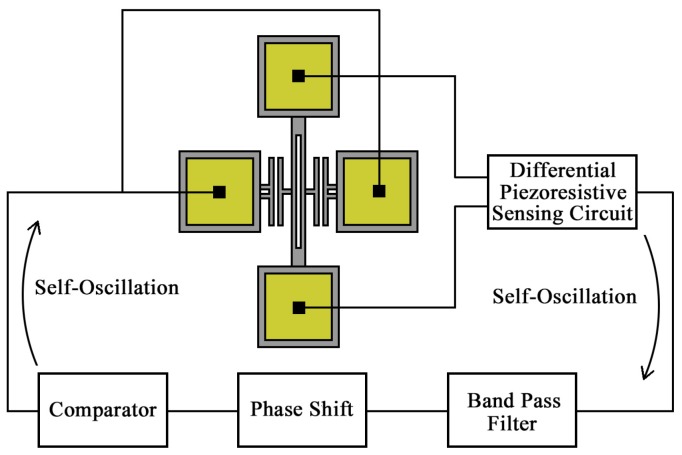
Micromechanical oscillator based on the DETF resonator.

The closed-loop tests are suitable for practical application field, and it is the first step of industrialization of MEMS resonant sensors. In the closed-loop tests, micromechanical oscillators based on the DETF resonators were built up as shown in Figure 10. The oscillation circuit contains a differential piezoresistive sensing circuit, a band pass filter, a phase shifter and a comparator. The DETF is electrostatically actuated in its anti-phase vibration mode and its transverse displacement is detected through the differential piezoresistive sensing method. The resulting signal is filtered and phase shifted before entering into comparator. The output signal of comparator together with a DC bias is applied on the driving electrodes to excite the DETF resonator. The time-varying feedback force compensates the energy dissipation and this closed loop feedback sustains the oscillation of DETF. The oscillation frequency changes as the sensor tilts, which ensures a practical measurement of tilt angle.

In the static test, the tilt sensor was mounted still on the vertical plane. The frequency output was recorded by a frequency counter for 20 min with a 10 ms gate time. A short gate time ensures frequency data being rapidly read-out, but also leads to a relatively high systematic noise caused by the frequency counter. Therefore, trade off between quick response and high stability should be considered in practical use.

**Figure 11:**
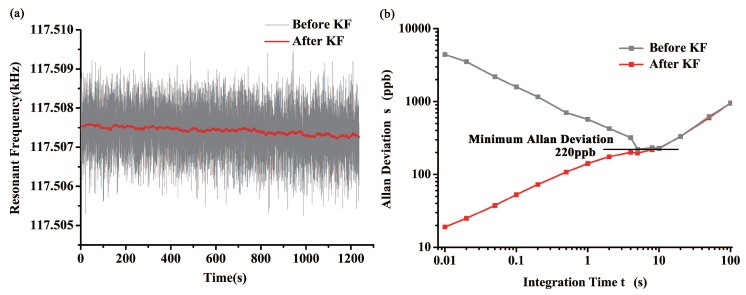
(**a**) Static frequency data of DETF B before and after Kalman Filter; (**b**) Allan deviation of the static frequency data with different parameter R.

The measured sensor signal is usually affected by different factors such as quantization noise, velocity random walk, bias instability, etc. [25]. As shown in Figure 11a, the output frequency of DETF B oscillator is very noisy and the frequency fluctuation is up to 3 Hz, which dramatically degrades the performance of tilt sensor. Kalman Filter (KF) was found to be an adequate way to reduce noise and improve resolution of the system. Comparing to moving-average filters, Kalman Filter enables the tilt sensor a better dynamic response and adaptability of complex testing environments [26]. In order to improve the filtering effect while reducing the delay time caused by KF, we have found the most appropriate model parameters in our previous work [2].

Allan deviation method was also used to study the frequency stability of oscillator circuit. As indicated in Figure 11b, KF can reduce the short-time noise remarkably as R increases. However, it is incapable of canceling long-term drift and accordingly Allan deviation of the processed data are the same as the integration time gets longer. Minimum Allan deviation of the origin data is found to be 220 ppb at 5 s, and the resolution of our tilt sensor can be got by:

(5) $$\begin{array}{c} R=\frac{A\times f_{0}}{S} \end{array}$$

where *A* is the minimum Allan deviation of the data, $f_{0}$ is the characteristic frequency of the DETF, *S* is the scale factor of the tilt sensor. In this way, resolution of the tilt sensor is $0.002{}^{\circ }$.

**Figure 12:**
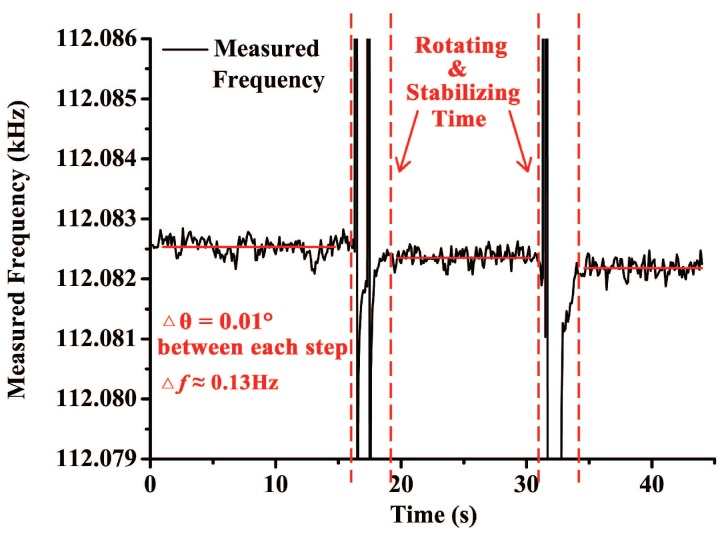
Resolution test of the tilt sensor.

Resolution of the sensor was also experimentally measured. Installation of the laboratory apparatus and the tilt sensor was the same as the open-loop test. DETF B was embedded in the self-oscillation circuit to read out its resonant frequency in real time while the rotary table was working. The sampling interval of the frequency data was 100 ms. Positional accuracy of the rotary table is $0.01{}^{\circ }$, so the minimum angle variation was set to be $0.01{}^{\circ }$. Figure 12 shows the performance of the tilt sensor in the resolution test. Frequency shift between each step was about 0.13 Hz, which coincide with the scale factor measured in the open-loop test. Response time of the sensor is measured to be about 2 s. As a result, it can be found that resolution of the tilt sensor is better than $0.01{}^{\circ }$.

Accuracy is another feature to evaluate the performance of the tilt sensor, which is measured as a highest deviation of a value represented by the sensor from the ideal or true value. It can be estimated by the following formula:

(6) $$\begin{array}{c} A=\sqrt{N^{2}+H^{2}+R^{2}} \end{array}$$

where *N* is the nonlinearity of the output, *H* is its hysteresis, and *R* is its repeatability. To calibrate these characteristics, DETF B was tested repeatedly in the range from $-30{}^{\circ }$ to $30{}^{\circ }$ with a step of $10{}^{\circ }$. During each step, the rotary table was still for 5 s and average of the frequency datum were recorded. Output of DETF B at each position is given in Table 2.

**Figure 13:**
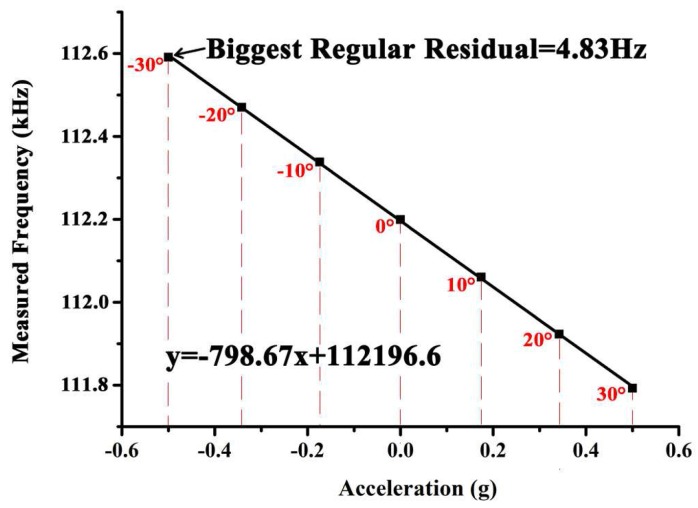
Linear fit of the frequency data.

After a linear fitting of the data as shown in Figure 13, transfer function of DETF B can be described as:

(7) $$\begin{array}{c} y=-798.67x+112196.6 \end{array}$$

where *y* is the frequency data, and *x* is the applied acceleration. Maximum regular residual was found to be 4.83 Hz at the position of −30°, which corresponds to −0.5 g. Thus the nonlinearity can be derived by:

(8) $$\begin{array}{c} N=\frac{Y_{Nmax}}{Y_{FS}}\times 100\%\;FS=\frac{4.83\;\mathrm{Hz}}{798.7\;\mathrm{Hz}}\times 100\%FS=0.61\%\;FS \end{array}$$

where $Y_{Nmax}$ is the maximum regular residual, $Y_{FS}$ is the full scale frequency shift, which in this experiment (±30°, which corresponds to ±0.5 g) is 798.7 Hz, FS represents full scale of the measurement range.

Hysteresis of the sensor represents the inconsistency of its output during loading and unloading. It can be seen from the experimental data that the maximum deviation is 3.5 Hz, at the position of −20°. Thus the hysteresis can be got by:

(9) $$\begin{array}{c} H=\frac{Y_{Hmax}}{2\times Y_{FS}}\times 100\%\;FS=\frac{3.5\;\mathrm{Hz}}{2\times 798.7\;\mathrm{Hz}}\times 100\%\;FS=0.22\%\;FS \end{array}$$

where $Y_{Hmax}$ is the maximum difference of the output.

The repeatability represents inconsistency of the measured results in multiple experiments. It can be derived by:

(10) $$\begin{array}{c} R=\frac{c\times S_{av}}{Y_{FS}}\times 100\%\;FS=0.46\%\;FS \end{array}$$

where *c* is the confidence factor, and in this four-back-and-forth experiment c = 3.182, $S_{av}$ is the standard deviation of the measured data.

To sum up, the nonlinearity of the tilt sensor is 0.61% FS, the hysteresis is 0.22% FS, and the repeatability is 0.46% FS. According to the fuluma above, accuracy of the tilt sensor is calculated to be 0.79% FS.

## 5. Conclusions

This paper reports a novel resonant MEMS tilt sensor of high sensitivity maintained in an expanded measurement range of $0{}^{\circ }$∼$360{}^{\circ }$. The tilt sensor is microfabricated using a standard SOI technology. Open loop testing results show that the presented sensor can achieve a sensitivity of 11.53 Hz/degree in the full scale range. Closed-loop oscillator circuit has been built up and characterized by using Allan deviation method. The testing results show that the resolution of the tilt sensor is 0.002°, and the accuracy is 0.79% FS. The proposed accelerometer was compared with others from publications in the measurement range, die area, scale factor, resolution and accuracy together. The comparison results were shown in Table 3. These values compare well with other reported MEMS tilt sensors.

Future work will focus on further increasing scale factor and resolution of the tilt sensor. Integrated packaging of the whole system will be the main content of the research. Also, compensation of temperature drift and other environmental factors will be studied.

## Figures and Tables

**Table 1:** The geometry dimensions of DETF resonators.

Resonant beam length	300 μm
Resonant beam width	3 μm
Device thickness	10 μm
Die area	5.5 mm × 5.5 mm
DETF A frequency	151.815 kHz
DETF B frequency	138.201 kHz
DETF C frequency	138.211 kHz

**Table 2:** Output of DETF B at each position.

Tilt Angle (degree)	Acceleration (g)	Frequency Data (Hz)					
		1st	2nd	3rd	4th	Average	Standard Deviation
−30	−0.5	112,592.6	112,592	112,590.3	112,589.6	112,591.1	1.2
−20	−0.342	112,470.7	112,469.4	112,473.1	112,468.7	112,470.5	1.6
−10	−0.174	112,337.9	112,337.6	112,340.3	112,337	112,338.2	1.2
0	0	112,198.4	112,199.9	112,202	112,198.9	112,199.8	1.4
10	0.174	112,059.4	112.061.1	112,061.6	112,060.5	112,060.7	0.8
20	0.342	111,921.7	111,923.4	111,924.3	111,923.8	111,923.3	1.0
30	0.5	111,791.2	111,793.2	111,793.8	111,793.3	111,792.9	1.0

**Table 3:** Comparison between this work and other reported.

	Range (degree)	Die Area (mm × mm)	Scale Factor	Resolution	Accuracy
Our work	±180	5.5 × 5.5	11.53 Hz/degree	$0.002{}^{\circ }$	0.79% FS
[4]	±20	4.5 × 4	24.5 Hz/degree	$0.00003{}^{\circ }$	/
[13]	±180	13 × 13	6.317 Hz/g	$0.76{}^{\circ }$	0.67% FS
[27]	±90	7 × 7	45 ppm/degree	/	/
